# Multi-TALK: Multi-Microphone Cross-Tower Network for Jointly Suppressing Acoustic Echo and Background Noise

**DOI:** 10.3390/s20226493

**Published:** 2020-11-13

**Authors:** Song-Kyu Park, Joon-Hyuk Chang

**Affiliations:** Department of Electronic Engineering, Hanyang University, Seoul 04763, Korea; thdrbwkd@hanyang.ac.kr

**Keywords:** acoustic echo suppression, noise suppression, attention mechanism, temporal convolutional network, cross-tower

## Abstract

In this paper, we propose a multi-channel cross-tower with attention mechanisms in latent domain network (Multi-TALK) that suppresses both the acoustic echo and background noise. The proposed approach consists of the cross-tower network, a parallel encoder with an auxiliary encoder, and a decoder. For the multi-channel processing, a parallel encoder is used to extract latent features of each microphone, and the latent features including the spatial information are compressed by a 1D convolution operation. In addition, the latent features of the far-end are extracted by the auxiliary encoder, and they are effectively provided to the cross-tower network by using the attention mechanism. The cross tower network iteratively estimates the latent features of acoustic echo and background noise in each tower. To improve the performance at each iteration, the outputs of each tower are transmitted as the input for the next iteration of the neighboring tower. Before passing through the decoder, to estimate the near-end speech, attention mechanisms are further applied to remove the estimated acoustic echo and background noise from the compressed mixture to prevent speech distortion by over-suppression. Compared to the conventional algorithms, the proposed algorithm effectively suppresses the acoustic echo and background noise and significantly lowers the speech distortion.

## 1. Introduction

As the demand for smart devices operated by speech commands continues to increase, the coupling between the loudspeaker and microphone in a smart device under ambient noise significantly degrades the quality of speech communication and the performance of automatic speech recognition. In particular, owing to the diversification of smart devices, it has become more challenging to reliably remove acoustic echo and background noise in various environments. The traditional approach to acoustic echo cancellation (AEC) is to estimate the acoustic echo path from the loudspeaker to the microphone using an adaptive filter [1]. To allow these AEC methods to work appropriately, several additional issues should be resolved. Common problems include the nonlinearity caused by the frequency response characteristics of the loudspeaker and divergence of the adaptive filter in a double-talk situation in which near-end speech and far-end echo coexist. To solve the nonlinearity problem caused by the loudspeaker, a residual echo suppression (RES) module that can remove the nonlinear echo can be designed separately [2,3]. In order to prevent the adaptive filter from divergence due to double-talk, a method of employing a separate double-talk detector was used [4,5] to force the adaptive filter not to update when a double-talk occurs. In addition, it is more challenging when both echo and noise exist, which further require a noise suppression module. In the case of noise suppression (NS), a method of separation using the statistical characteristics of noise and speech was applied [6,7], and AEC and NS modules were serially combined [8,9]. Although several algorithms have been designed by combining AEC and NS modules, the divergence of an adaptive filter is still caused by background noise, near-end speech, changes in the acoustic echo path, and nonlinear distortion. Therefore, a more sophisticated method for obtaining high-quality speech is still needed. In addition, these serial connections are likely to change the statistical characteristics of the noise under the influence of AEC and cause speech distortion owing to continuous suppression. Therefore, it is difficult to balance echo and noise attenuation without near-end speech distortion because acoustic echo cancellation and noise reduction adversely affect each other.

In recent years, various methods and structures using sophisticated nonlinear modeling have been applied to the field of speech signal processing, showing high performance in fields such as speech enhancement, source separation, reverberation cancellation, and AEC. With AEC applications, in particular, a fully connected network (FCN) was introduced to estimate the optimal RES gain as a separate module for removing nonlinear residual echoes [10]. In [11], the background noise and acoustic echo were continuously removed using a stacked deep neural network (DNN) model applying an FCN, although the phase of the near-end speech could not be modeled effectively because only the magnitude of the short-time Fourier transform (STFT) coefficients was used.A convolutional recurrent network was recently proposed in [12], and the complex spectrum of the near-end speech was directly estimated using the complex spectrum of the mixture and far-end signal. However, there was a drawback of requiring a separate near-end speech detector. To solve this problem, a time domain based network with an end-to-end structure was recently proposed and demonstrated better results than the aforementioned conventional STFT domain algorithms. In the field of source separation, in particular, a fully convolutional time domain audio separation network (Conv-TasNet) [13] has been adopted as a state-of-the-art solution in speech separation. As a method to overcome the disadvantages of the frequency domain based algorithm, a dilated convolution operation that can view a wide range of time series was applied, and the phase of the signal was implicitly modeled as the advantage of the end-to-end structure. However, from the viewpoint of suppressing noise or acoustic echo, since the scale-invariant speech-to-distortion ratio (SI-SDR) that does not consider scale was used a loss function in [13], which leads to speech distortion, this structure should be modified.

Inspired by the success of Conv-TasNet in the field of source separation, we propose a multi-channel cross-tower with attention mechanisms in latent domain network (Multi-TALK). Conv-TasNet [13], which operates in the time domain, was originally designed for audio and speech separation, but is modified for acoustic echo and background noise suppression. In the proposed Multi-TALK, the main contributions of the proposed approach are threefold. First, we change the structure of the separator [13] into a cross-tower structure and design each tower to estimate the echo and noise that need to be removed. Each tower uses a temporal convolution network (TCN) block to maintain the advantages of the Conv-TasNet and adds a latent loss to avoid speech distortion by using the outputs of each tower at the decoder stage. Second, an auxiliary encoder is added to extract the far-end features, and an attention mechanism is applied to convey the effective far-end features to the cross-tower network. In addition, the encoder is replaced with a parallel encoder to enable an expansion to multi-channel inputs. Finally, instead of directly estimating speech from the mixture as in [13], the echo and noise estimated from the cross-tower are removed from the compressed mixture before being decoded to estimate the near-end speech in the latent domain. Another set of attention mechanisms is also applied to prevent speech distortion. The proposed approach is evaluated in terms of objective measures related to speech quality and shows a significant improvement over several conventional algorithms.

Section 2 describes the proposed system, which is composed of an encoder, a cross-tower, and a decoder. The dataset, model architecture, evaluation metrics, and numerical results are described in Section 3. Finally, some concluding remarks are provided in Section 4.

## 2. Proposed Multi-Channel Cross-Tower with Attention Mechanisms

### 2.1. Signal Modeling

**Figure 1:**
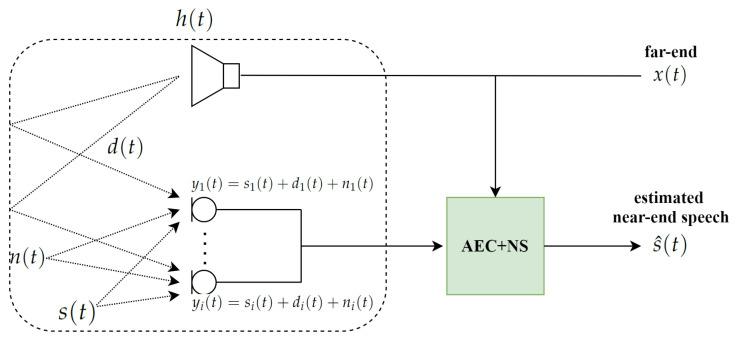
Block diagram of suppressing acoustic echo and background noise in the multi-channel scenario. AEC, acoustic echo cancellation; NS, noise suppression.

The basic concept of a near-end speech estimation when acoustic echo and background noise coexist is depicted in Figure 1. The *i*-th microphone observation $y_{i}\left(t\right)$ consists of a near-end signal $s_{i}\left(t\right)$, acoustic echo $d_{i}\left(t\right)$, and background noise $n_{i}\left(t\right)$, satisfying the following:

(1) $$y_{i}\left(t\right)=s_{i}\left(t\right)+d_{i}\left(t\right)+n_{i}\left(t\right),$$

where *t* is a time index. The acoustic echo $d_{i}\left(t\right)$ is a signal that has nonlinear distortion caused by the loudspeaker and returns to the microphone array through a room impulse response (RIR). The purpose of Multi-TALK is to remove acoustic echo $d_{i}\left(t\right)$ and background noise $n_{i}\left(t\right)$ from the microphone input mixtures $y_{i}\left(t\right)$ for estimating the clean near-end speech $s_{i}\left(t\right)$.

### 2.2. Overview of Multi-TALK

**Figure 2:**
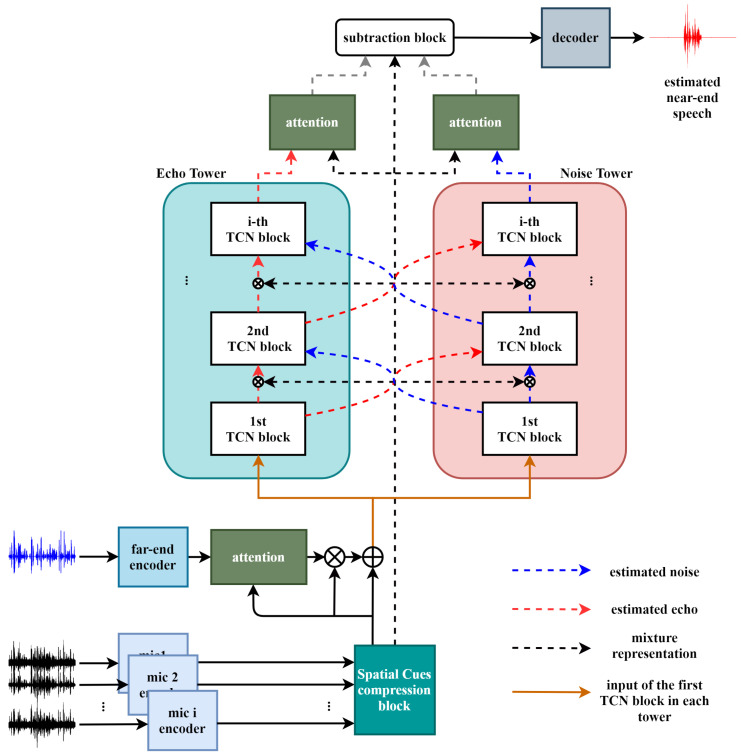
Block diagram of the proposed multi-channel cross-tower with attention mechanisms in latent domain network. TCN, temporal convolution network.

This section describes the proposed Multi-TALK algorithm, which is depicted in Figure 2. Multi-TALK consists of three parts: an encoder, a cross-tower, and a decoder. The first part of the network is the encoder part, which effectively converts the time domain waveform into its latent features. It is designed to effectively change the multi-channel mixtures and far-end information into latent features and pass this to the input of the cross-tower network. The second part is the cross-tower network, which is the main part of the proposed algorithm. Each tower repeatedly estimates echo and noise separately while exchanging the latent features from the neighboring tower through TCN blocks.Finally, the decoder section is designed to reconstruct the near-end speech using three types of information in the latent domain: the estimated echo and noise through each tower and the compressed mixture of microphones. The details of each part of the proposed algorithm are described in the following subsections.

### 2.3. Two Different Encoders with an Attention Mechanism

**Figure 3:**
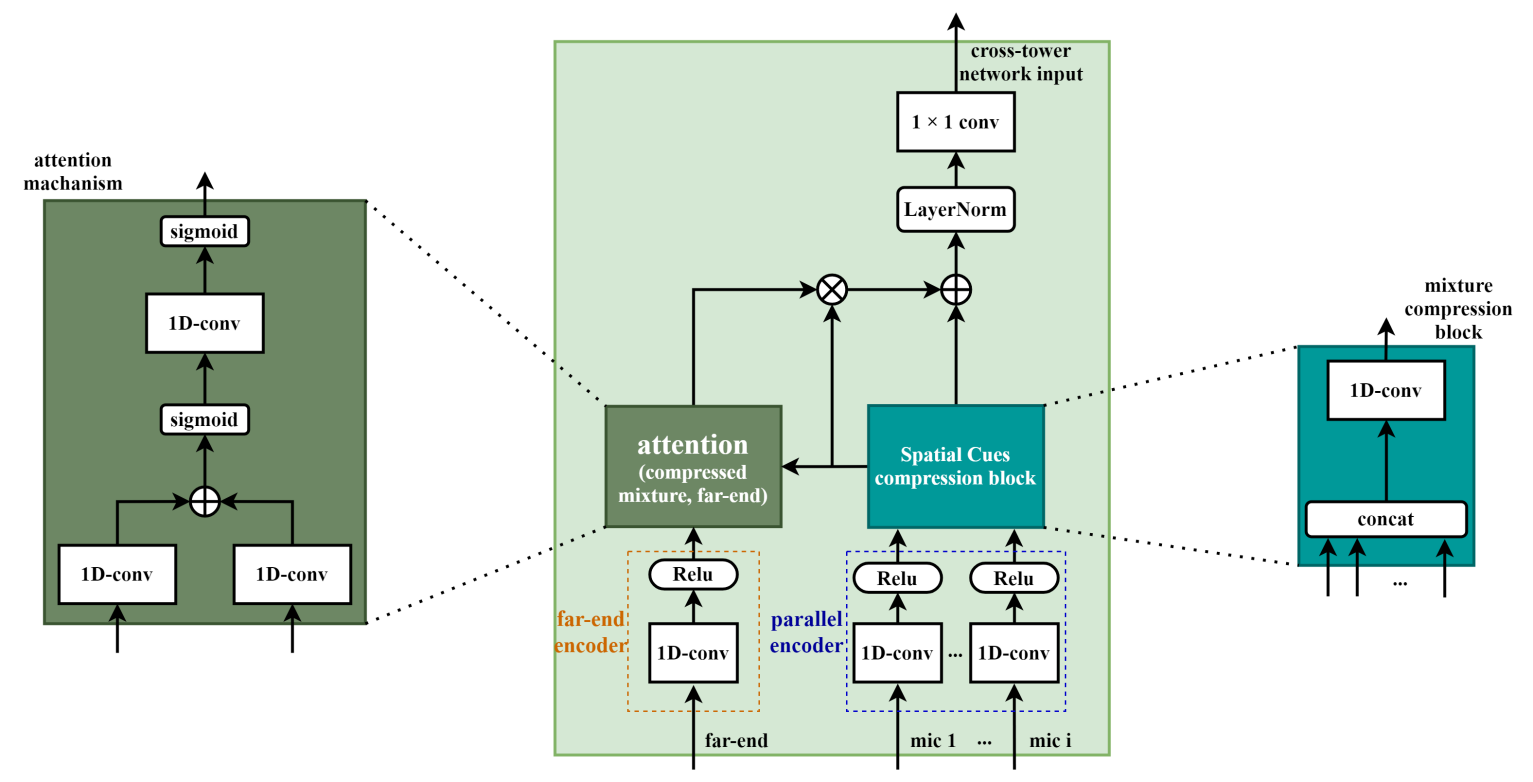
Mic encoders and auxiliary encoder with an attention mechanism.

The input mixture for each microphone can be divided into overlapping *L*-length segments denoted by $y_{i,k}$$\in R^{1\times L}$, where *k* = 1, 2,.., $\hat{T}$, and $\hat{T}$ represents the total number of segments in the input signal. Each microphone input mixture, $y_{i,k}$, is converted to an *N*-dimensional representation $w_{i}$, by applying a 1D convolution operation, which is represented by a matrix multiplication:

(2) $$\begin{array}{cc} \; & w_{i}=H\left(y_{i,k}U_{i}\right), \end{array}$$

where $U_{i}$$\in R^{L\times N}$ contains *N* vectors (the basis functions of each encoder) of length *L* for transforming the waveform to latent features, and H(·) is the rectified linear unit [14,15] to ensure that the feature is non-negative. Unlike single-channel processing, spatial information can be used as additional features when multiple microphones are available. Spatial cues between multi-channel signals such as inter-channel time difference (or inter-channel phase difference) and inter-channel level difference can indicate the location of the speech source. These spatial characteristics have been shown to be particularly beneficial when combined with spectral characteristics over the frequency domain in several fields, such as source separation, speech enhancement, and voice activity detection [16,17,18,19,20,21,22,23]. Unfortunately, these spatial features are typically extracted in the frequency domain using STFT, making it difficult to integrate perfectly using the time domain method. Therefore, in the proposed algorithm, the spatial information of multi-channel mixtures is extracted using a parallel encoder, which is independently trained for each mixture to be utilized for network information. As shown in Equation (2), this parallel encoder contains *N* convolution kernels for each microphone mixture $y_{i,k}$. This parallel encoder operates similarly to the STFT, but is non-deterministic, as it is determined by learning. However, it was revealed from [24] that the learned filters of the encoders are similar to the auditory system. After each microphone mixture passes through the parallel encoder, the spatial cues between the microphones are compressed through a 1D convolution operation. Therefore, multi-channel mixtures are converted to a compressed mixture of the same dimension as the single-channel process as follows:

(3) $$w_{x}=H\left(concat\left[w_{1},w_{2},...,w_{m}\right]U_{x}\right),$$

where $U_{x}$$\in R^{mN\times N}$ and *m* and $w_{x}$ are the total number of microphones and a representation of the compressed multi-channel mixture obtained through a parallel encoder and a 1D convolution operation, respectively. In addition, the far-end information, which helps to subtract an acoustic echo, is extracted by passing through the auxiliary encoder, and it is transmitted as compressed latent features of the mixtures through an element-wise multiplication. To build a more effective connection, an attention mechanism [25] is applied to enable an efficient information delivery, as described in detail in Figure 3. The latent features of the far-end and compressed multi-channel mixture are mapped to the intermediate feature space. The dimension of the intermediate feature is the same as the dimensions of the two input channels, and the kernel size is set to one. By applying another 1D convolution operation on top of the two intermediate features of the same dimension, we create a mask that can only extract highly correlated latent features from the two inputs. The mask is then expressed through the following formula:

(4) $$\begin{array}{c} \begin{array}{cc} B_{w_{x},w_{f}} & =\sigma (L_{W_{x}}+L_{w_{f}}),\\ M_{encoder-attention} & =\sigma (L_{B_{w_{x},w_{f}}}), \end{array} \end{array}$$

where $w_{f}$ and σ(·) denote a representation of far-end obtained through an auxiliary encoder and the sigmoid function, respectively. In addition, $L_{\left\{\cdot \right\}}$ is the output of a 1D convolution operation applied to {·}. By using this mask, information of the far-end highly correlated with the latent features of compressed mixture is passed only to the network input.

### 2.4. TCN Blocks in the Cross-Tower

Motivated by Conv-TasNet [13], we design a cross-tower network consisting of two towers, unlike the existing network that directly estimates near-end speech by modifying the connection of TCN blocks. Each tower contains TCN blocks composed of *R* layers, and each layer contains *X* number of 1D convolution blocks with dilation factors of 1, 2, 4, … 2${}^{X-1}$. The dilation factors can be increased exponentially to ensure a large temporal context window compared to the frequency domain convolution network to effectively model the time dependencies of the speech signal. In the cross-tower network, more accurate latent features of the echo and noise are estimated as more iterations of the TCN block proceed. To this end, both outputs from the tower and neighboring tower at the previous iteration are used for the compressed mixture at the current iteration. For example, in the echo tower, the latent features of the echo are first multiplied with those of the mixture, which remain the highly correlated latent features with the echo only. However, in the obtained result, the residual noise components can also be included, which should be subtracted by using the latent variables of the noise at the previous iteration. The noise tower is also similarly explained. This is to use the estimated echo and noise as weights and biases, which is motivated by the adaptation layer of speakerbeam [26,27]. In other words, the previous TCN blocks of each tower act as auxiliary networks that provide the weights and biases to the compressed mixture re-entered in the next TCN blocks. As a result, in the latent domain, the compressed mixture is transformed according to the mission of each tower at each iteration and is offered as intermediate inputs to further improve the performance.

**Figure 4:**
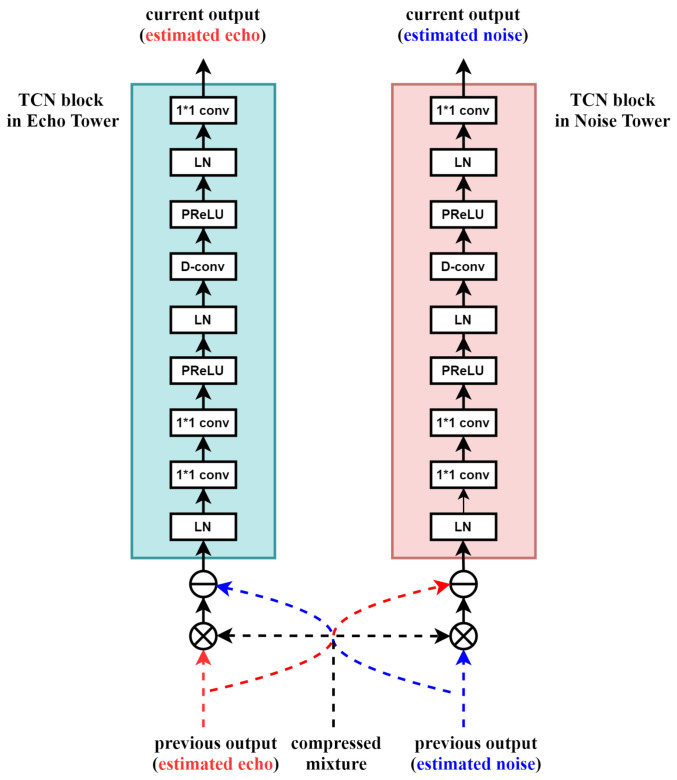
TCN block in the cross-tower network.

The operation conducted in the TCN block, depicted in Figure 4, is as follows: The TCN blocks normalize the intermediate input features and perform a 1 × 1 convolution operation. Because the first TCN blocks of each tower have no outputs from the previous TCN blocks, only a compressed mixture with an attention mechanism between the compressed mixture and the far-end is used. The following operations are 1 × 1 convolution operations as a bottleneck layer and depth-wise convolution with parametric rectified linear units [28] followed by another normalization block. Global normalization is performed in all normalization blocks. The last 1×1 convolution operation is used to create the same number of channels as the compressed mixture.

### 2.5. Near-End Speech Estimation in the Latent Domain with Attention Mechanisms Applied

Similar to the inverse STFT, the decoder is a transposed convolutional layer that inverts the *w*$\in R^{1\times L}$ representation back into a time domain waveform:

(5) $$\tilde{y}=wV,$$

where $\tilde{y}$ is a reconstruction of *y* and the rows of *V*$\in R^{N\times L}$ are the decoder basis functions, each of which is *L* in length. The superimposed reconstructed segments are summed to produce the final waveform. The near-end speech representation estimated in Conv-TasNet [13] is calculated by applying the mask *m*$\in R^{1\times N}$ to the mixture representation *w*:

(6) D=w⊙m,

where ⊙ denotes an element-wise multiplication. The waveform of the near-end speech $\tilde{s}$ is reconstructed by the decoder such that:

(7) $$\tilde{s}=DV.$$

Instead of estimating speech by multiplying the mask to the mixture representation in Equation (6), to utilize the cross-tower structure, the near-end speech is estimated in the latent domain using the estimated echo and noise.

Therefore, it is possible to estimate more accurate near-end speech by learning to be represented as close to near-end speech as possible while only near-end speech-related information remains in the latent domain before decoding processing. If the echo and noise estimated from each tower are simply removed from the compressed mixture representation, the possibility of speech distortion is still high. To prevent this, the attention mechanisms are applied. As used in the encoder, the compressed mixture representation and the estimated echo and noise are mapped separately through a 1D convolution operation in the intermediate feature space. In addition, each creates two masks that can extract the latent features only, which are highly correlated with the echo and noise.

(8) $$\begin{array}{cc} m_{echo-attention} & =\sigma (L_{B_{w_{x},{\hat{d}}_{R}}}),\\ m_{noise-attention} & =\sigma (L_{B_{w_{x},{\hat{n}}_{R}}}),\\ B_{w_{x},{\hat{d}}_{R}} & =\sigma (L_{W_{x}}+L_{{\hat{d}}_{R}}),\\ B_{w_{x},{\hat{n}}_{R}} & =\sigma (L_{W_{x}}+L_{{\hat{n}}_{R}}), \end{array}$$

where ${\hat{d}}_{R}$ and ${\hat{n}}_{R}$ are representations of the *R*-th TCN output of the echo and noise tower. Although the general attention emphasizes the corresponding part through the mask, the attention mechanisms used before the decoder are applied to subtract the highly correlated echo and noise from the compressed mixture representation to estimate the near-end speech. Therefore, Equation (6) is transformed into the following:

(9) $$D=w_{x}-w_{x}m_{echo-attention}-w_{x}m_{noise-attention}.$$

**Figure 5:**
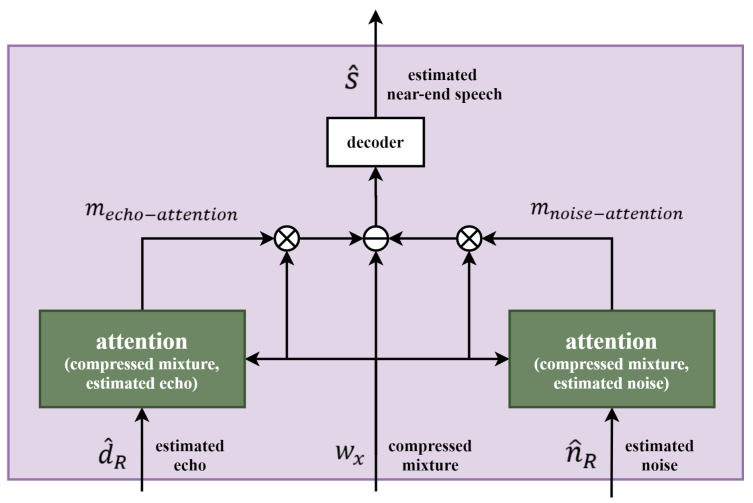
Decoder and mask for estimating near-end speech with attention mechanisms.

The reason for applying the attention mechanisms is to prevent speech distortion that occurs when the echo and noise are over-suppressed. The above process is depicted in Figure 5.

### 2.6. Training Objective

In Multi-TALK, the loss function is represented by several weighted sums. First, the main loss is the speech-to-distortion ratio (SDR) [29,30] for estimating near-end speech in the time domain. The negative SI-SDR used for source separation [13] does not take into account scaling errors. On the other hand, this negative SDR has the advantage of preserving the scale and matching the mixture [31]. The SDR equation is expressed as follows:

(10) $$SDR=10\log_{10}\left(\frac{\left|\right|s_{target}{\left|\right|}^{2}}{\left|\right|s_{target}-\hat{s}{\left|\right|}^{2}}\right),$$

where ∥·∥ denotes the $\ell_{2}$-norm function and $S_{target}$ is the near-end speech at the first microphone used in this study. Second, the logarithmic mean squared error (LMSE) [32] is defined as an additional loss in the latent domain to reduce the error of the echo and noise repeatedly estimated by the TCN blocks in each tower.

(11) $$LMSE_{P}=10\log_{10}(|d-{\hat{d}}_{P}{|}^{2})+10\log_{10}(|n-{\hat{n}}_{P}{|}^{2}),$$

where *d*, *n*, ${\hat{d}}_{P}$, and ${\hat{n}}_{P}$ are the latent features of the target echo, the latent features of the target noise, the *P*-th TCN output of the echo tower, and the *P*-th TCN output of the noise tower, respectively. Combining these two types of loss, the total loss is expressed as follows:

(12) $$\begin{array}{cc} Loss_{total} & =\frac{-\alpha_{M}*SDR+\left(1-\alpha_{M}\right)\frac{1}{4}\sum_{P=1}^{R}q^{R-P}[10\log_{10}(|d-{\hat{d}}_{P}{|}^{2})+10\log_{10}(|n-{\hat{n}}_{P}{|}^{2})]}{\alpha_{M}+\left(1-\alpha_{M}\right)\frac{1}{4}\sum_{P=1}^{R}\left(q^{R-P}+q^{R-P}\right)}\\ \; & =\frac{-\alpha_{M}*SDR+\left(1-\alpha_{M}\right)\frac{1}{4}\sum_{P=1}^{R}q^{R-P}LMSE_{P}}{\alpha_{M}+\left(1-\alpha_{M}\right)\frac{1}{2}\sum_{P=1}^{R}q^{R-P}}, \end{array}$$

where *R* is the number of TCN blocks in each tower, *q* is set to $\frac{1}{2}$, and $\alpha_{M}$ is set to 0.7. Even if the TCN blocks increase infinitely at each tower, the weighted sum of additional loss does not exceed the weight of the SDR by multiplying $\frac{1}{4}$ with the additional loss.

## 3. Experiments and Simulation Results

### 3.1. Dataset

Simulations were conducted under various conditions to evaluate the performance of the proposed Multi-TALK algorithm. Based on the TIMITdatabase [33] sampled at 16 kHz, near-end and far-end speakers were separately selected and grouped into 100 pairs (25 male-female, 25 female-male, 25 male-male, and 25 female-female). There are 10 utterances per speaker in the TIMIT database, and thus, we concatenated 3 randomly selected utterances of the same speaker to generate a far-end signal. In addition, one-hundred pairs were randomly selected from Musan [34] to generate a music far-end, whose duration is equal to that of the speech far-end generated from the TIMIT database. To simulate the nonlinear acoustic echo signal from the microphone through the power amplifier, loudspeaker, and acoustic echo path, three processes were executed [10], i.e., a hard clipping of the loudspeakers, the application of a nonlinear simulation model, and an acoustic echo signal through the RIR [35]. These are described in further detail below. In the first step, we applied artificial hard clipping [36] using the following:

(13) $$x_{\mathrm{hard}}\left(t\right)=\left\{\begin{array}{cc} -x_{\max}, & x\left(t\right)<-x_{\max}\\ x(t), & \left|x\left(t\right)\right|\leq x_{\max}\\ x_{\max}, & x\left(t\right)>x_{\max}, \end{array}\right.$$

where $x_{\mathrm{hard}}$ denotes the output of hard clipping, and $x_{\max}$, the maximum value of |x(t)|, was set to 0.8. Next, we employed memoryless sigmoidal nonlinearity [37] to the far-end signal to mimic the nonlinear properties of the loudspeakers as follows:

(14) $$x_{nl}\left(t\right)=\gamma \left(\frac{2}{1+\exp(-\alpha \times \beta (t))}-1\right),$$

where:

(15) $$\beta \left(t\right)=1.5\times x_{\mathrm{hard}}\left(t\right)-0.3\times x_{\mathrm{hard}}^{2}\left(t\right).$$

The value of the sigmoid function γ was set to 4, and α=4 if β(t) > 0 and α = $\frac{1}{2}$ otherwise.

**Figure 6:**
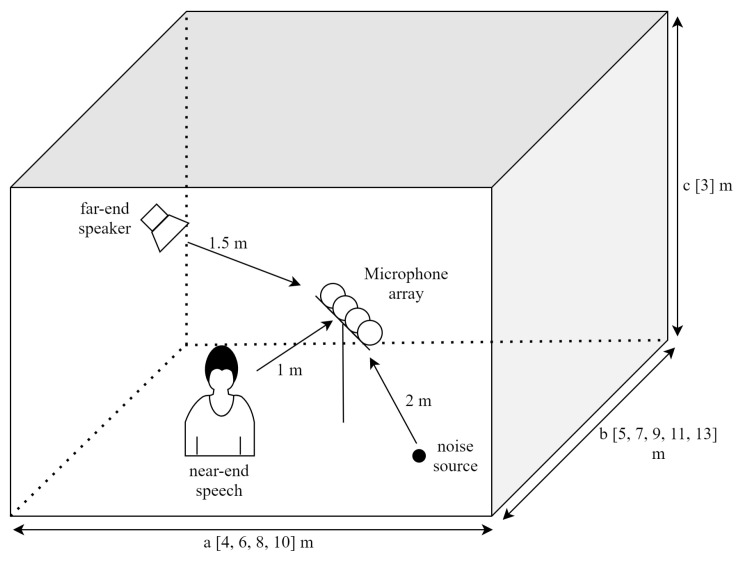
Simulation environment.

For near-end speech, one utterance, randomly chosen per speaker, was extended by padding zeros with the same length as the paired signal at the far-end. Furthermore, twelve different types of noise (i.e., Volvo and white from the NOISEX-92database [38], street and traffic from ITU-Trecommendation P.501 [39], babble, cafeteria, living room, metro, office, restaurant, station, and vacuum from the MS-SNSDdatabase [40]) were used for the training, validation, and testing. Six types of noise (cafeteria, metro, office, station, traffic, vacuum) were used for training, and the remaining six noises (babble, living room, restaurant, street, Volvo, white) were used for testing. To simulate the real environment, we modeled 20 different rooms of dimensions *a* × *b* × *c* m^3^, where a=[4,6,8,10], b=[5,7,9,11,13], and c=3. To generate RIRs from each sound source to the microphone array, the microphones were linearly arranged in the center of the room, maintaining a distance of 5 cm between the neighboring ones. The length of an RIR from each sound source to the microphone array was set to 512, and the reverberation time (RT60) was chosen randomly from the set [0.2 s, 0.3 s, 0.4 s]. Even with the same room size and RT60, in order to generate RIRs in various locations, three sound sources (far-end speaker, near-end speech, and noise) were generated at random positions maintaining distances of 1.5 m, 1 m, and 2 m from the microphone array to create a total number of 600 different RIR sets. Of these, five-hundred RIR sets were used for learning, and the remaining 100 RIR sets were used for testing. The simulation environment is shown in Figure 6.

Finally, a signal-to-echo ratio (SER) level of [-6 dB, -3 dB, 0 dB, 3 dB, 6 dB] for near-end speech and an acoustic echo signal was randomly selected, and the adjusted acoustic echo signal was mixed with the near-end speech. In addition, one of the six noises was chosen arbitrarily, and the noise scaled by a randomly selected signal-to-noise ratio (SNR) of [0 dB, 4 dB, 8 dB, 12 dB] was added to the mixed signal. The measured SER and SNR under a double-talk scenario are defined as follows:

(16) $$SER=10\log_{10}\frac{E[s^{2}\left(t\right)]}{E[d^{2}\left(t\right)]},$$

(17) $$SNR=10\log_{10}\frac{E[s^{2}\left(t\right)]}{E[n^{2}\left(t\right)]},$$

where E[·] denotes the statistical expectation operation. Similar to the preparation of the training database, we created a test database using RIR sets and each sound source not used for training. The test database was also generated by randomly selecting the SER and SNR from [-4 dB, -2 dB, 0 dB, 2 dB, 4 dB] and [3 dB, 6 dB, 9 dB]. Through the above process, a total of 7000 samples for training and 800 samples for testing were generated. Half of the acoustic echoes used in each set were speech, and the other half were music.

### 3.2. Model Architecture

As mentioned in Section 2, the proposed Multi-TALK is a variant of the Conv-TasNet implementation. Similar hyperparameters of the model as in [13] were set to *N* = 256, *L* = 40, *B* = 256, *H* = 128, *P* = 3, *X* = 4, and *R* = 4. In the case of *L*, because 20 was used in the 8 kHz database, forty was used in the 16 kHz database in the same context. The Adam optimizer [41] was used, and the learning rate was fixed at 1e−4. The network was trained for 100 epochs, and if the validation loss did not improve within three epochs, an early pause was applied. In addition, a 50% stride size was used in the convolutional encoder.

### 3.3. Evaluation Metrics

Each of the conventional algorithms for evaluation was implemented based on the stacked DNN [11], CRN [12], and original Conv-TasNet [13] among the existing echo and noise suppression algorithms. Conv-TasNet and its modified algorithms, including Multi-TALK, were implemented as non-causal systems. The performance of these algorithms was compared with that of the proposed method. Four objective measures were used for performance evaluation. The perceptual evaluation of the speech quality (PESQ) [42], short-time objective intelligibility (STOI) [43], and SDR were used to evaluate the double-talk periods in which near-end and far-end speech exist simultaneously and were evaluated using the echo return loss enhancement (ERLE) [36,44] in single-talk periods where only far-end speech occurred. The ERLE, which indicates the degree of echo cancellation, is defined as follows:

(18) $$ERLE=10\log_{10}\frac{E[y^{2}\left(t\right)]}{E[{\tilde{s}}^{2}\left(t\right)]},$$

where a high ERLE score means a high level of echo cancellation in the single-talk periods. In the double-talk periods, a high PESQ (between -0.5 and 4.5), STOI (between 0 and 1), and SDR indicate improved speech quality and intelligibility.

### 3.4. Numerical Results

Table 1 and Table 2 show the average PESQ, ERLE, STOI, and SDR scores of each algorithm when processing a total of 800 (400/400) test mixtures using noise, RIR sets, and acoustic echo that were not used in the learning.

We compared the performance of the stacked DNN that sequentially suppresses noise and echo, the CRN using spectral mapping in the frequency domain, Conv-TasNet, variants of Conv-TasNet, and the proposed Multi-TALK algorithm. Algorithms other than the CRN did not use a near-end detector, and thus, the evaluation was conducted without a near-end detector. The highest performance score for each measurement is highlighted in bold. Most of the results showed that all tested algorithms provided improved performance compared to performance without processing. In the case of Conv-TasNet, when the SI-SDR was used as a training objective, the ERLE and SDR scores, which consider scale as important, decreased compared to when using the SDR. Besides, when the auxiliary encoder was used with the attention mechanism, it was possible to observe the improvement in acoustic echo cancellation performance in particular. A total of four objective evaluations were conducted on the conventional algorithms, stacked DNN [11], CRN [12], and Conv-TasNet [13] and its modifications. For a fair evaluation with other algorithms, a single-channel version of the proposed Multi-TALK was added. Compared with conventional algorithms, the single-channel version of Multi-TALK showed better performance in terms of all of the considered objective measurements. In addition, comparing Table 1 and Table 2, even when the results were compared according to the echo type, the proposed algorithm exhibited superior performance relative to other algorithms when both speech and music echo types were applied. Particularly in the case of the proposed Multi-TALK, in which spatial cues can be compressed and used, increasing the number of microphones improved almost all performance scores. Furthermore, an experiment was conducted when noise and near-end speech were present without acoustic echo, and three objective measures excluding ERLE were measured in the period where the near-end existed. The results are shown in Table 3. The far-end created by using music or concatenating utterances rarely had silent periods. Therefore, the duration of near-end speech and noise without echo was relatively shorter than the duration of those with echo in the mixture used for learning. Thus, the evaluation in environments where echo does not exist resulted in over-suppression and low SDR. Especially, unlike the method of estimating the masks limited to a specific range, the CRN, which directly estimates the spectrum of near-end speech, showed poor results in all measures because of estimation error. Furthermore, the auxiliary encoder configured separately for echo cancellation did not effectively increase the SDR because it provides unnecessary information to the network. However, unlike the SDR, which is simply calculated using the difference in time domain signals, the PESQ or STOI showed different results because it underwent complex processes such as filtering and equalizing to calculate only the parts related to auditory perception. Since the proposed algorithm had a module that separately processed information related to echo estimation, Table 3 shows meaningful results in all measures even when acoustic echo was not present.

In addition, to confirm the robustness of the proposed algorithm, we performed an evaluation of mismatch datasets. For this, evaluation datasets were created by changing the parameters of Equations (13) and (14). The value of $x_{\max}$ was randomly selected from 0.4 to 0.7 in units of 0.1, and if β(t) > 0, the value of α was set between one and five and between 0.1 and 0.9 otherwise. Another set of evaluation datasets was generated closer to the real environment by changing the RIR length from 512 to 2048. Although the overall performance in Table 4 and Table 5 is degraded by the mismatch environments, this indicates that the proposed algorithm works reliably in different conditions.

**Figure 7:**
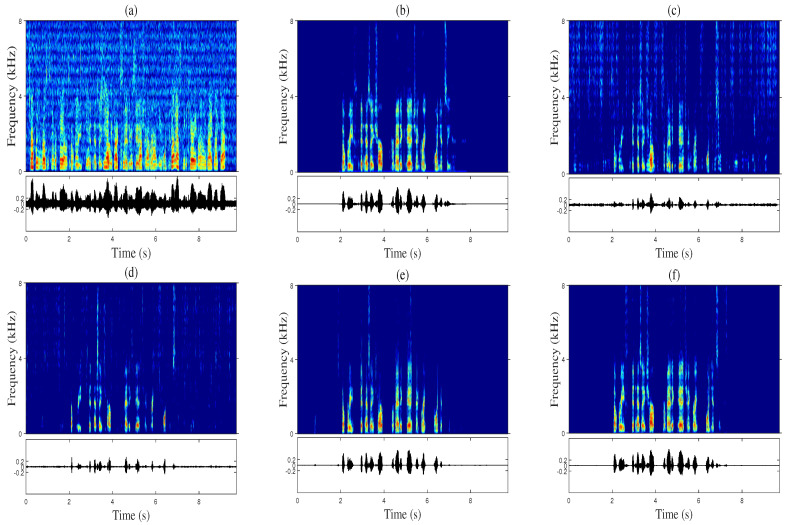
Spectrogram (top) and waveform (bottom) comparison under white noise and speech-type at the far-end (at a signal-to-echo ratio (SER) of -4 dB and an SNR of 3 dB): (**a**) microphone input signal, (**b**) near-end speech, (**c**) stacked DNN [11], (**d**) CRN [12], (**e**) single-channel version of Multi-TALK, and (**f**) the proposed Multi-TALK (4 ch).

**Figure 8:**
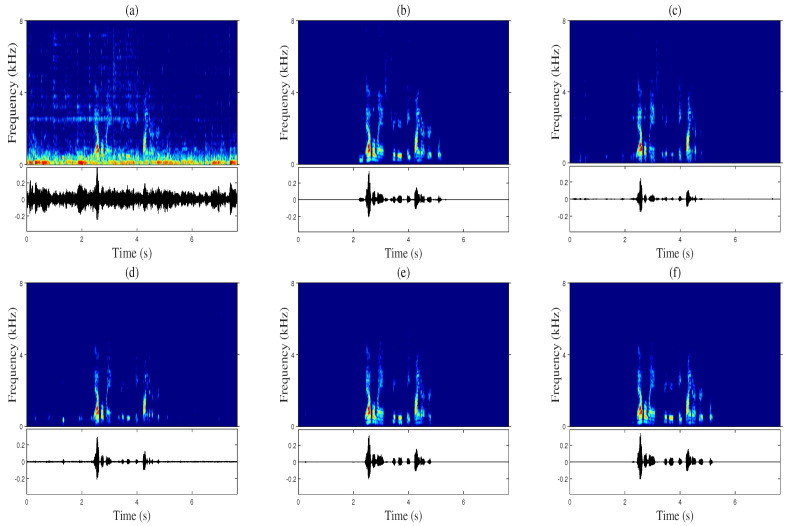
Spectrogram (top) and waveform (bottom) comparison in babble noise and the music-type far-end (at an SER of -4 dB and an SNR of 6 dB): (**a**) microphone input signal, (**b**) near-end speech, (**c**) stacked DNN [11], (**d**) CRN [12], (**e**) single-channel version of Multi-TALK, and (**f**) the proposed Multi-TALK (4 ch).

Figure 7 and Figure 8 show a comparison of the spectrogram and waveform for the enhanced speech of randomly selected audio samples in the test. As shown in Figure 7 and Figure 8, the proposed Multi-TALK suppresses acoustic echo most effectively compared to other algorithms during single-talk periods while showing the lowest speech distortion during periods of double talk.

However, the single-channel version of Multi-TALK (e) in Figure 7 and Figure 8 caused speech distortion in the spectrogram, but compared with other measures, the STOI scores were measured relatively high. This was because in the process of scoring the STOI, the low-frequency bands, which are sensitive to hearing, were calculated for frames with 40 dB less power than the frame with the largest power. Since Multi-TALK using multiple microphones significantly reduced speech distortion, the proposed Multi-TALK demonstrated superiority over conventional algorithms in terms of speech quality and acoustic echo and background noise suppression performance. In all previous experiments, the proposed algorithm showed the best performance compared to conventional single-channel algorithms when expanded to multiple channels that can utilize the spatial information of a microphone array.

## 4. Conclusions

In this study, we proposed the Multi-TALK algorithm, which simultaneously suppresses acoustic echo and background noise. Rather than estimating near-end speech directly from the mixture, Multi-TALK is designed to use a cross-tower for estimating the echo and noise to be removed and then uses these estimates for near-end speech estimation. To this end, the loss function is added to estimate the echo and noise, and attention mechanisms are used for effective learning and improved performance. Experiments based on multiple datasets showed that the proposed approach with spatial information achieved outstanding performance when compared with the conventional methods in terms of multiple objective speech quality measures.

## Figures and Tables

**Table 1:** Average perceptual evaluation of the speech quality (PESQ), short-time objective intelligibility (STOI), and speech-to-distortion ratio (SDR) under double-talk and the average echo return loss enhancement (ERLE) under single-talk where the far-end was a speech signal. TasNet, time domain audio separation network; Multi-TALK, multi-channel cross-tower with attention mechanisms in latent domain network.

Algorithm	PESQ	ERLE	STOI	SDR
unprocessed	1.60	-	0.527	−2.1
stacked DNN [11]	1.81	24.73	0.590	4.6
CRN [12]	1.83	23.36	0.577	5.0
Conv-TasNet [13]	1.73	23.43	0.624	−7.7
Conv-TasNet + SDR loss	1.79	27.70	0.631	6.3
Conv-TasNet + SDR loss + auxiliary encoder	1.83	26.77	0.656	7.5
Conv-TasNet + SDR loss + auxiliary encoder (attention)	1.87	30.80	0.682	8.0
Multi-TALK (1 ch)	1.94	32.62	0.690	8.1
Multi-TALK (2 ch)	2.43	41.09	0.801	10.4
Multi-TALK (4 ch)	**2.50**	**45.78**	**0.811**	**11.0**

**Table 2:** Average PESQ, STOI, and SDR under double-talk and the average ERLE under single-talk where the far-end was a music signal.

Algorithm	PESQ	ERLE	STOI	SDR
unprocessed	1.65	-	0.547	−1.8
stacked DNN [11]	1.86	25.97	0.598	4.4
CRN [12]	1.93	24.85	0.588	5.5
Conv-TasNet [13]	1.78	23.32	0.626	−7.9
Conv-TasNet + SDR loss	1.82	25.47	0.630	7.0
Conv-TasNet + SDR loss + auxiliary encoder	1.83	28.12	0.646	7.8
Conv-TasNet + SDR loss + auxiliary encoder (attention)	1.86	30.04	0.666	8.0
Multi-TALK (1 ch)	1.90	31.61	0.669	8.2
Multi-TALK (2 ch)	2.31	38.00	0.730	9.9
Multi-TALK (4 ch)	**2.34**	**43.94**	**0.771**	**10.6**

**Table 3:** Average PESQ, STOI, and SDR for the near-end single-talk period.

Algorithm	PESQ	STOI	SDR
unprocessed	2.28	0.748	5.5
stacked DNN [11]	2.31	0.644	3.8
CRN [12]	1.31	0.549	5.0
Conv-TasNet [13]	2.45	0.778	−8.8
Conv-TasNet + SDR loss	2.45	0.777	11.6
Conv-TasNet + SDR loss + auxiliary encoder	2.36	0.709	4.6
Conv-TasNet + SDR loss + auxiliary encoder (attention)	2.47	0.796	12.3
Multi-TALK (1 ch)	2.47	0.795	12.6
Multi-TALK (2 ch)	2.60	0.820	12.6
Multi-TALK (4 ch)	**2.61**	**0.826**	**12.7**

**Table 4:** Average PESQ, STOI, and SDR under double-talk and the average ERLE under single-talk where the far-end was a speech signal with nonlinearity mismatch.

Algorithm	PESQ	ERLE	STOI	SDR
unprocessed	1.62	-	0.529	−2.1
stacked DNN [11]	1.80	23.57	0.580	4.3
CRN [12]	1.21	19.83	0.410	2.8
Conv-TasNet [13]	1.63	22.14	0.609	−8.3
Conv-TasNet + SDR loss	1.66	28.73	0.604	6.1
Conv-TasNet + SDR loss + auxiliary encoder	1.70	27.29	0.628	7.2
Conv-TasNet + SDR loss + auxiliary encoder (attention)	1.79	29.03	0.658	7.5
Multi-TALK (1 ch)	1.86	30.19	0.662	7.7
Multi-TALK (2 ch)	2.13	29.93	0.716	8.5
Multi-TALK (4 ch)	**2.17**	**33.38**	**0.725**	**8.9**

**Table 5:** Average PESQ, STOI, and SDR under double-talk and the average ERLE under single-talk where the far-end was a music signal with room impulse response (RIR) mismatch.

Algorithm	PESQ	ERLE	STOI	SDR
unprocessed	1.71	-	0.503	−2.0
stacked DNN [11]	1.88	24.84	0.570	4.1
CRN [12]	1.22	23.84	0.349	1.8
Conv-TasNet [13]	1.47	19.17	0.534	−8.0
Conv-TasNet + SDR loss	1.51	24.16	0.536	5.9
Conv-TasNet + SDR loss+auxiliary encoder	1.70	28.89	0.593	7.1
Conv-TasNet+SDR loss+auxiliary encoder (attention)	1.67	26.70	0.591	6.8
Multi-TALK (1 ch)	1.73	26.23	0.598	7.0
Multi-TALK (2 ch)	1.79	29.53	0.627	6.9
Multi-TALK (4 ch)	**1.89**	**30.51**	**0.636**	**7.2**
